# Use of Extrapolation in New Drug Approvals by the US Food and Drug Administration

**DOI:** 10.1001/jamanetworkopen.2022.7958

**Published:** 2022-04-19

**Authors:** Daniel Feldman, Jerry Avorn, Aaron S. Kesselheim

**Affiliations:** 1 Program on Regulation, Therapeutics, and Law, Division of Pharmacoepidemiology and Pharmacoeconomics, Department of Medicine, Brigham and Women’s Hospital, Harvard Medical School, Boston, Massachusetts; 2 Rowan University School of Osteopathic Medicine, Stratford, New Jersey

## Abstract

**Importance:**

The US Food and Drug Administration (FDA)–approved indications can be factors in prescribing practices and insurance coverage, yet the frequency with which the extrapolation of clinical characteristics from pivotal trial data to the final approved indication occurs is not well understood.

**Objectives:**

To evaluate the frequency of extrapolation beyond pivotal trial data into approved indications in relation to disease severity, disease subtype, and concomitant medication use.

**Design, Setting, and Participants:**

In a cross-sectional study, the characteristics of patients in pivotal trials of 105 novel drug approvals from 2015 to 2017 were identified and compared with the FDA-approved indications for the drugs. Main sources analyzed included FDA reviews, published material describing the pivotal trials, and the original drug labeling. The study was conducted from July 4, 2019, to June 1, 2021.

**Exposures:**

Clinical characteristics of pivotal trials used in FDA approval.

**Main Outcomes and Measures:**

Main outcomes included the nature and frequency of extrapolation from study populations to the final indications. Extrapolation was defined as the granting of an indication for use in a broader population than was included in the pivotal trials on the basis of disease severity, disease subtype, or concomitant medication use.

**Results:**

Among the 105 novel FDA drug approvals studied, 23 extrapolations of trial population characteristics to the approved indication were identified in 21 drugs (20%): 12 times (29%) in 2015, 3 times (15%) in 2016, and 6 times (14%) in 2017. Extrapolation of trial findings to patients with greater disease severity was most common (n = 14 drugs), followed by differences in disease subtype (n = 6) and concomitant medication use (n = 3).

**Conclusions and Relevance:**

The findings of this study suggest that extrapolation from pivotal trial data to FDA-approved indications is common. Although extrapolations may be grounded in reasonable clinical predictions, they can limit the generalizability of such indications to specific prescribing decisions; these findings suggest a greater need for close postapproval monitoring to determine whether new safety issues arise, or effectiveness differs from expectations when these medications are used in broader real-world populations.

## Key Points

**Question:**

How often does the US Food and Drug Administration extrapolate the findings of a drug’s pivotal trial data to its approved indication, in terms of disease severity, subtype, or use of concomitant medication?

**Findings:**

In this cross-sectional study of 105 new drugs approved from 2015 to 2017, extrapolation beyond pivotal trial participants to other patient populations was noted 23 times in 21 drug approvals. Extrapolation of disease severity was most common, followed by disease subtype and concomitant medication use.

**Meaning:**

The findings of this study suggest that extrapolation by the US Food and Drug Administration beyond pivotal trial data to final indications is common, highlighting the need for close postapproval monitoring.

## Teaser

This cross-sectional study examines extrapolation of data for US Food and Drug Administration (FDA) drug approval based on disease severity, disease subtype, and concomitant medication use.

## Introduction

The US Food and Drug Administration (FDA) approves a new prescription drug if its benefits are judged to outweigh its risks in pivotal clinical trials^1^; the FDA and manufacturers then define the approved indications for the drug based on evidence from the trials. The indication is registered in drug labeling to guide prescribing and promotional statements. It can also help establish the contours of insurance coverage.^2^ However, pivotal trials leading to FDA approval are necessarily limited in the populations they cover.^3^ Although attention has been given to limitations in the demographic diversity of the trial population,^4,5^ less attention has been paid to whether a narrowly defined clinical trial population is then used as the basis for extrapolation of trial findings to a broader population of patients than was represented in those trials. Controversially, the FDA initially approved aducanumab in 2021 for all patients with Alzheimer disease, even though it was studied only in those with mild disease.^6^

Extrapolation from pivotal trials to a broader approved indication may be appropriate when there is no basis for expecting a different outcome in the wider postapproval population.^7^ However, extrapolation can also lead to different clinical outcomes in practice than were observed in the pivotal trials. Such differences were seen on a large scale with the approval of spironolactone for heart failure based on the Randomized Aldactone Evaluation Study when, after publication of the trial, spironolactone prescription rates in routine practice increased by a factor of 5 to include a substantially older population than was included in the initial trial. Inclusion of these individuals led to a 3-fold increase in mortality associated with hyperkalemia in patients given the drug, far higher than was seen in the original clinical trials.^8,9^ This increase was due in part to differing populations being prescribed spironolactone in practice, as well as concomitant medication use and eligibility criteria compared with those used in the original trial.^9^

To our knowledge, no systematic analysis of such extrapolation in FDA approvals has been published; however, several meta-analyses and trials discussed herein have reported differences in outcomes when a drug is used in patients with disease severity, disease subtypes, or concomitant medication use that differed from those studied in the pivotal trials. In a study of patients with rheumatoid arthritis included in trials of tumor necrosis factor inhibitors compared with patients receiving the drug in routine practice, the latter individuals had substantially lower disease severity than those eligible for the trials and experienced less clinical benefit.^10^ Use of corticosteroids occurred in 44% to 69% of the trial participants, compared with 29% to 54% of those seen in routine care.^11^ This higher concurrent corticosteroid use during the trial could have also contributed to the differing outcomes seen in the 2 settings. Differences in disease subtype can also influence outcomes.^12^ A study of treatments for non–small cell lung cancer found that improved accuracy of histologic categorization would result in more appropriate prescribing and outcomes in routine care.^13^ Yet extrapolation of findings from a pivotal trial that predominantly included 1 histologic subtype to all histologic subtypes of non–small cell lung cancer could influence patients’ response to that treatment. This pattern has been observed across many diseases.^14,15,16^

To determine how frequently extrapolation occurs from pivotal trial populations to final FDA-approved indications, we analyzed a cohort of FDA drug approvals to determine how 3 key clinical characteristics of patients in the pivotal trials were represented in the approved indications: disease severity, disease subtype, and concomitant medication use.

## Methods

### Data Sources and Extraction

We identified 113 new molecular entity and biologic drug approvals after pivotal trials from 2015 to 2017, using the FDA website,^17^ and reviewed the approved indications on the original drug labeling. We then used Section 7 of the FDA Summary Reviews to identify the pivotal trials for each medication (ie, those submitted to the FDA to support the drug’s approval). Additional resources included the FDA statistical review, published articles on pivotal trials, AccessMedicine, and ClinicalTrials.gov. The study was conducted from July 4, 2019, to June 1, 2021. This study followed Strengthening the Reporting of Observational Studies in Epidemiology (STROBE) reporting guideline for cross-sectional studies and used public, nonidentifiable data that did not constitute human participants research. This study was exempt from institutional review board approval and the need for informed consent in accordance with 45 CFR §46.102.

Key information analyzed for each approval included pivotal trial inclusion and exclusion criteria, distribution of trial participants by disease subtype, disease severity, use of concomitant drugs, and other patient characteristics, and the resulting original approved indication. We excluded 7 products because of our inability to analyze data from all pivotal trials, as well as 1 drug approved based on animal studies alone.

### Data Analysis

We studied extrapolation of findings from trial participants to approved indications across 3 domains: disease severity, subtypes of disease treated, and concomitant medication use. We compared a drug’s pivotal trial characteristics with the FDA-approved indications. Extrapolation was defined as the granting of an indication for use in a broader population than was included in the pivotal trials on the basis of disease severity, disease subtype, or concomitant medication use. Underrepresentation or skew toward 1 end of a spectrum of disease severity, disease subtype, or concomitant medication use was also considered extrapolation. More than 1 category of extrapolation could be related to each approved indication.

Instances of extrapolation were systematically identified by one of us (D.F.) and analyzed by the team thereafter. In addition, one of us (A.S.K.) independently analyzed 20% (n = 21) of the 105 approvals. The approvals were selected randomly by assigning a number from 1 to 105 and using a random number generator to select the sample. The findings from the sample analysis were discussed to ensure consistency and reproducibility.

To assess disease severity, we examined the scales or criteria used to classify, include, or exclude patients from a trial based on disease activity or severity. For example, the trials for secukinumab to treat psoriasis and other conditions included only patients with a modified investigators' global assessment score of 3 to 4, indicating moderate to severe plaque psoriasis. This limited population was reflected in the language of the approved indication. If the indication had not specified moderate to severe, we would have considered the indication to be based on extrapolation because the trial did not include patients with lower disease severity. Similarly, extrapolation was designated if the trial population was heavily skewed toward one side of the spectrum of disease severity but was not indicated on the formal indication.

To assess disease subtypes, we determined the range of variations or subtypes for a given condition and compared those included in the pivotal trials with those in the approved indication. For example, patients in the secukinumab trial were included only if they had plaque-type psoriasis; the indication reflects this, as the drug is indicated for use only in patients with this diagnosis. If, however, the indication had not specified plaque psoriasis, we would have considered this to be extrapolation because the trial did not include patients with any other psoriasis subtype. If trials were heavily skewed toward one subtype of disease that was not reflected in the official indication, that would also represent extrapolation. Heavily skewed, in most cases, was defined as 5% or less of the study population.

To assess concomitant medication use, we determined whether trial participants were receiving additional relevant medications and whether that concomitant therapy was reflected in the approved indication. For example, extrapolation would be designated if patients in a trial of secukinumab were using a topical corticosteroid along with the study drug, but the combination was not included in the indication. Investigators, however, prohibited the use of topical corticosteroids during the trial, which aligns with the approved indication for secukinumab. Data analysis was performed using Microsoft Excel, Version 16.58, (Microsoft Corp).

## Results

There were 113 novel drug approvals in the cohort; of these, we were able to extract relevant information for 105 drugs (93%). These included 42 drugs approved in 2015, 20 in 2016, and 43 in 2017 (Table 1). The most common FDA drug review divisions with the most new drug approvals were oncology (20 [18%]), hematology (14 [12%]), and gastroenterology and inborn errors (12 [11%]). Of the approvals analyzed, we identified 23 instances of extrapolation from characteristics of patients in the trials to approved indications based on disease severity, disease subtype, or concomitant medication use in 21 drugs (20%). This occurred 12 times (29%) in 2015, 3 times (15%) in 2016, and 6 times (14%) in 2017. The results are summarized in Table 2.

**Table 1.** Sample Derivation and Types of Extrapolation Identified in the Cohort

Variable	No. (%)			
	2015	2016	2017	Total
Drugs	45 (40)	22 (19)	46 (41)	113 (100)
FDA				
Approvals analyzed	42 (93)	20 (91)	43 (93)	105 (93)
Approvals that used extrapolation	12 (29)	3 (15)	6 (14)	21 (20)
Concomitant medication^a^	2	1	0	3
Disease^a^				
Severity	7	2	5	14
Subtype	5	0	1	6

Abbreviation: FDA, US Food and Drug Administration.

^a^ Extrapolation was identified in more than 1 of the non–mutually exclusive categories in 2 approvals in 2015. These findings were considered separate instances of extrapolation, bringing the total to 23 cases across 21 approvals.

**Table 2.** Details of Extrapolation Related to Disease Severity, Disease Subtype, and Concomitant Medication Use in New Drug Approvals

Drug	Approval		Extrapolation	
	Year	Indication	Nature	Category
Edoxaban	2015	To reduce the risk of stroke and systemic embolism in patients with nonvalvular atrial fibrillation and for treatment of deep vein thrombosis and pulmonary embolism following 5-10 d of initial therapy with a parenteral anticoagulant	77.8% Of pivotal trial participants had a CHADS_2_ score of 2 or 3	Disease severity
Ceftazidime with avibactam	2015	For complicated urinary tract infections including pyelonephritis caused by the following susceptible microorganisms: *Escherichia coli*, *Klebsiella pneumoniae*, *Citrobacter koseri*, *Enterobacter aerogenes*, *Enterobacter cloacae*, *Citrobacter freundii*, *Proteus* species, and *Pseudomonas aeruginosa* in patients aged ≥18 y	Several pathogens included in the indication accounted for 0.4%-4.6% of pathogens in the experimental arm (*Pseudomonas aeruginosa*, *Citrobacter freundii*, *Citrobacter koseri*, *Proteus mirabilis*, and *Enterobacter cloacae*)	Disease subtype
Cholic acid	2015	Bile acid synthesis disorders due to single enzyme defects and as an adjunctive treatment of peroxisomal disorders, including Zellweger spectrum disorders, in patients who exhibit manifestations of liver disease, steatorrhea, or complications from decreased fat-soluble vitamin absorption	55% Of patients in the pivotal trial had 3β-hydroxysteroid dehydrogenase single-enzyme defects or Zellweger syndrome; other single-enzyme defects occurred in as few as 1 participant	Disease subtype
Ivabradine	2015	Reduce the risk of hospitalization for worsening heart failure in patients with stable, symptomatic chronic heart failure with left ventricular ejection fraction ≤35%, who are in sinus rhythm with resting heart rate ≥70 beats/min, and either are receiving maximally tolerated doses of β-blockers or have a contraindication to β-blocker use	79% Of participants in the experimental group were also taking ACE inhibitors	Concomitant medication
			2% Of trial participants had heart failure class IV	Disease severity
Sacubitril with valsartan	2015	Reduce the risk of cardiovascular death and hospitalization for heart failure in patients with chronic heart failure and reduced ejection fraction	75.9% Of trial participants had heart failure class I or II; 0.8% had heart failure class IV	Disease severity
		Sacubitril with valsartan is usually administered in conjunction with other heart failure therapies in place of an ACE inhibitor or angiotensin II receptor blocker		
Brexpiprazole	2015	Adjunctive therapy to antidepressants for the treatment of major depressive disorder and schizophrenia	Mean PANSS scores ranged from 93.3 to 96.3, denoting a markedly ill to severely ill patient population	Disease severity
Rolapitant	2015	In combination with other antiemetic agents for the prevention of delayed nausea and vomiting associated with initial and repeat courses of emetogenic cancer chemotherapy, including, but not limited to, highly emetogenic chemotherapy	Main pivotal trial tested efficacy in highly emetogenic chemotherapies	Disease severity
Cariprazine	2015	Schizophrenia and acute treatment of manic or mixed episodes associated with bipolar I disorder	Mean (SD) PANSS score was 96.5 (9), indicating on average a severely ill patient population	Disease severity
Aripiprazole lauroxil	2015	Schizophrenia	Mean (SD) PANSS score was 92 (10.2), indicating on average a markedly ill patient population	Disease severity
Osimertinib	2015	Metastatic epidermal growth factor receptor T790M mutation positive non–small cell lung cancer when progressed during or after epidermal growth factor receptor–tyrosine kinase inhibitor therapy	96% Of patients had adenocarcinoma subtype of non**–**small cell lung cancer	Disease subtype
Alectinib	2015	Anaplastic lymphoma kinase-positive, metastatic non–small cell lung cancer that has progressed or patients are intolerant to crizotinib	96% Of patients had adenocarcinoma subtype of non–small cell lung cancer	Disease subtype
Patiromer	2015	A potassium binder indicated for the treatment of hyperkalemia	Exclusively tested in patients with hyperkalemia due to chronic kidney disease	Disease subtype
			All patients were taking renin-angiotensin-aldosterone system inhibitors	Concomitant medication
Obeticholic acid	2016	Primary biliary cholangitis in combination with UDCA in adults with an inadequate response to UDCA or as monotherapy in adults unable to tolerate UDCA	Mean (SD) Mayo risk score 4.2 (1.2), indicating, on average, a low risk of death in patients with primary biliary cholangitis who did not receive a transplant; >98% of participants’ scores fell into the lowest risk class	Disease severity
Eteplirsen	2016	Duchenne muscular dystrophy in patients who have a confirmed mutation of the DMD gene that is amenable to exon 51 skipping	Eligibility criteria required corticosteroid use	Concomitant medication
Nusinersen	2016	Spinal muscular atrophy in pediatric and adult patients	Spinal muscular atrophy subtype 4 not included in the pivotal trials	Disease severity
Telotristat	2017	Carcinoid syndrome diarrhea in combination with somatostatin analogue therapy in adults inadequately controlled by somatostatin analogue therapy	Patients with Karnofsky score <60 excluded from the trial	Disease severity
Ocrelizumab	2017	Relapsing or primary progressive forms of multiple sclerosis	Patients with greater Expanded Disability Status Scale scores due to multiple sclerosis were excluded; mean scores were low (2.75-2.86) in the relapsing trials	Disease severity
Edaravone	2017	ALS	Pivotal trial efficacy demonstrated in subset of patients diagnosed within 2 y of symptom onset and had FVC >80% and ALS Functional Rating Scale score >2	Disease severity
Emicizumab	2017	Routine prophylaxis to prevent or reduce the frequency of bleeding episodes in adult and pediatric patients with hemophilia A (congenital factor VIII deficiency) with factor VIII inhibitors	94% Of adult participants had the most severe form of hemophilia A	Disease severity
Netarsudil	2017	Reduction of elevated intraocular pressure in patients with open-angle glaucoma or ocular hypertension	Patients with high intraocular pressure (>26 mm Hg) excluded from the trial	Disease severity
Angiotensin II	2017	Increased blood pressure in adults with septic or other distributive shock	80.7% of participants had sepsis as the cause of distributive shock	Disease subtype

Abbreviations: ACE, angiotensin-converting enzyme; ALS, amyotrophic lateral sclerosis; DMD, Duchenne muscular dystrophy; FVC, forced vital capacity; PANSS, Positive-Negative Syndrome Scale; UDCA, ursodeoxycholic acid.

Extrapolation on the basis of disease severity occurred most frequently (n = 14). For example, edaravone was approved to treat amyotrophic lateral sclerosis (ALS). The underlying evidence for approval was based on a post hoc analysis of a study reporting that a subset of patients with ALS with shorter time since onset and less severe disease appeared to respond better to edaravone than to placebo. The subsequent pivotal trial was then designed with eligibility criteria including patients who had good functional status (forced vital capacity >80%, Amyotrophic Lateral Sclerosis Functional Rating Scale–Revised score ≥2) and were within 2 years of diagnosis. The FDA-approved indication, however, does not note this limitation and makes no distinction between potentially differing responses depending on disease duration or severity.

Extrapolation on the basis of disease subtype occurred in 6 instances. In the case of angiotensin II, more than 80% of participants in the clinical trial presented with confirmed septic shock as the cause of their vasodilatory shock. An additional 9.7% were categorized as having suspected sepsis as the cause of their shock. Vasodilatory shock can result from several distinct conditions, including anaphylaxis, pancreatitis, sepsis, and neurogenic shock. Despite not including all subtypes of shock in the trial, angiotensin II was indicated for increasing blood pressure in septic or other vasodilatory shock.

Extrapolation of indications based on the use or nonuse of concomitant medications was the least common (n = 3). For example, patients in the pivotal trial for ivabradine for heart failure were also receiving a variety of other treatments to manage this condition. However, the approved indication refers only to β-blockers, which were being used by 89% of patients given the study drug; the label does not refer to concurrent use of angiotensin-converting enzyme inhibitors, which were being used by 79% of patients receiving the study drug.

## Discussion

Using data from 105 novel FDA drug approvals from 2015 to 2017, this cross-sectional study sought to define instances of clinically meaningful extrapolation of indications beyond the characteristics of the pivotal trials on which drug approval was based. With this information, we found that extrapolation occurred in 20% of new drug approvals. We noted that such extrapolation is common and describe its prevalence in approved indications of many new drugs.

Some of the extrapolations we identified may be clinically plausible, but others could have implications for the effectiveness and safety of drugs when used in accordance with the FDA-approved indication. In some cases, the FDA acknowledges the presence of extrapolation in its review materials. For example, in the approval of edaravone for ALS, for which the pivotal trial showed varying efficacy based on disease severity, the FDA concluded that the disease has a variable course and it may not be possible to identify a stage at which treatment benefit may or may not be achieved. However, only trials that excluded patients with more severe disease found statistically significant and clinically relevant results for the drug compared with placebo. Even with this broad indication, some private insurers have chosen to tailor their coverage to the more-specific group of patients with ALS in whom it was most clearly shown to be effective^18^; public insurers may not have such flexibility. The initial list price of edaravone was $1410 per 60-mg infusion, or more than $189 000 for the first year of treatment.

Divergence between a regulator and payor can sow confusion and create conflict between clinicians, patients, and insurers if the FDA grants a drug an indication for a given condition, a physician prescribes it for a patient with that indication, and an insurer denies coverage by applying more restrictive criteria that it considers to be more in line with the clinical trial evidence. Conversely, if there is no such restriction on the part of the payor, covering the cost of an expensive drug such as edaravone for treatment of ALS in populations in which it is technically indicated by the FDA but has no demonstrated clinical benefit can result in higher health care spending without improved outcomes.

Use of a medication in routine practice often requires extrapolation beyond the exact patient population included in preapproval trials. For example, it may be reasonable to extrapolate efficacy data for angiotensin II from the type of vasodilatory shock included in the pivotal clinical trial to all types of vasodilatory shock. However, in other cases, extrapolation may be more questionable, as in the example of edaravone, for which disease severity had a clear association with efficacy. In the case of disease subtypes, not all patients may respond to treatment if disease subtypes vary in their course of progression.

Because it is impossible to precisely define all circumstances in which extrapolation is appropriate, robust postmarketing studies are needed to measure the outcomes associated with new medications in routine care. Such real-world observational studies can help determine how these therapies are being used as well as their usefulness in these untested populations.^19^ If a drug’s performance is found to differ in real-world populations owing to this extrapolation, the indication should be revised or a boxed warning added.^20^ In some circumstances, it might be appropriate for the FDA to require postapproval clinical trials or registries to clarify such issues, without delaying availability of the product. However, although 2007 legislation gave the agency the right to require such postmarketing investigation, such studies are often delayed or not completed.^21,22^

### Limitations

This study has limitations. The analysis was limited to 3 years of new drug approvals and was based on publicly accessible data. We therefore did not have insight into any nonpublished confidential data or discussions between the FDA and manufacturers and were unable to calculate descriptive statistics that were not included in the sources reviewed. Similarly, we were unable to identify whether all subtypes of a disease being treated were included in pivotal trials if the accessible data did not include the breakdown within participants. This level of access, however, simulates the access clinicians would have to guide their prescribing should they explore the data behind the approval.

Another limitation of this study is that, owing to the variability between trials and therapy targets, we could not apply a consistent definition for extrapolation across all the drugs. In addition, this study was not designed to investigate the clinical outcomes associated with the extrapolations identified, which should be the subject of future clinical trials and observational studies.

## Conclusions

Extrapolation of a drug’s indications beyond information available from the pivotal trials on which approval was based is often necessary in the approval of new medications because preapproval trials cannot possibly cover all patient subpopulations, age categories, and comorbidities. However, extrapolation of clinically limited data to generate broad official indications can sometimes extend past the bounds of what is plausible given the characteristics of patients studied in preapproval trials, potentially influencing the drug’s effectiveness and safety when used in routine practice. Such problems of nonrepresentativeness have been described in terms of patient age, sex, and race and ethnicity. To our knowledge, this is the first study to document the frequency of such extrapolation in terms of the clinical features of the disease being treated such as severity, subtype, and concomitant medication use. Although such extrapolation may be justified at the time of regulatory approval, our findings also point to the importance of follow-up research to confirm the expected outcomes. It may be beneficial to incorporate formal postapproval surveillance, using both prospective trials and well-conducted observational studies, into the rollout of novel therapies for which such extrapolation has occurred to ensure better ascertainment of real-world effectiveness and safety. When such clinical extrapolation is necessary at the time of approval, its details should be made clear in the labeled indications for physicians and patients. Until that time, it would be useful for physicians to recognize that the FDA-approved indication alone may be insufficient information on which to decide whether a given medication will benefit a patient who may differ meaningfully from those studied in the clinical trials on which approval was based.
